# Motilin fluctuations in healthy volunteers determined by liquid chromatography mass spectrometry

**DOI:** 10.3389/fendo.2024.1348146

**Published:** 2024-03-13

**Authors:** Rachel E. Foreman, Christopher A. Bannon, Richard G. Kay, Frank Reimann, Fiona M. Gribble

**Affiliations:** Institute of Metabolic Science, Addenbrooke’s Hospital, Cambridge, United Kingdom

**Keywords:** motilin, GIP, insulin, CCK, fasting, post-prandial, metabolism, LC-MS/MS and/or quantitative mass spectrometry

## Abstract

**Introduction:**

Motilin is a hormone secreted by specialised enteroendocrine cells in the small intestine, and is known to modulate gastrointestinal motility in humans, regulating the migratory motor complex. It is understudied at least in part due to the lack of commercially available immunoassays.

**Method:**

A multiplexed liquid chromatography mass spectrometry (LC-MS/MS) method was optimised to measure motilin, insulin, C-peptide, GIP (1–42) and GIP (3–42). Corresponding active ghrelin concentrations were determined by immunoassay. Ten healthy volunteers with no prior history of gastroenterological or endocrine condition attended after overnight fast and had blood samples taken every 15 minutes for 4 hours whilst continuing to fast, and then further sampling for 2 hours following a liquid mixed meal. Hunger scores were taken at each time point using a visual analogue scale. Normal bowel habit was confirmed by 1 week stool diary.

**Results:**

Motilin levels fluctuated in the fasting state with an average period between peaks of 109.5 mins (SD:30.0), but with no evidence of a relationship with either ghrelin levels or hunger scores. The mixed meal interrupted cyclical motilin fluctuations, increased concentrations of motilin, insulin, C-peptide, GIP(1-42) and GIP(3-42), and suppressed ghrelin levels.

**Discussion:**

This study highlights the utility of LC-MS/MS for parallel measurement of motilin alongside other peptide hormones, and supports previous reports of the cyclical nature of motilin levels in the fasting state and interruption with feeding. This analytical method has utility for further clinical studies into motilin and gut hormone physiology in human volunteers.

## Introduction

Motilin (MLN) is a hormone from the upper small intestine, which was originally identified as a peptide stimulating gastric motility (1). Recent studies have suggested that motilin additionally plays a role in signalling hunger and food reward, re-igniting research into this peptide (2–4). Clinical interest in motilin includes the long standing use of erythromycin, an antibiotic with motilin receptor (MLNR) agonist activity, to treat intestinal dysmotility conditions such as gastroparesis (5, 6).

Motilin is one of around 20 gut hormones that arise from specialised enteroendocrine cells (EECs) in the intestinal epithelium. In the duodenum, populations of EECs produce hormones such as glucose-dependent insulinotropic polypeptide (GIP), cholecystokinin (CCK) and secretin, which are secreted following food ingestion and coordinate nutrient digestion, absorption and disposition (7). Motilin is secreted by a distinct population of duodenal enteroendocrine cells known as M-cells which co-express ghrelin (8, 9). Unlike motilin, however, the major reserves of ghrelin are in the stomach, where their release is suppressed following feeding (10). Motilin physiology is relatively understudied because neither *Mln* nor *Mlnr* are thought to be expressed in rodents (11), although a recent manuscript described an analgesic role for both in the spinal cord of rats (12).

Profiles of motilin secretion are somewhat different from those of other gut hormones. Motilin release is typically associated with phase III of the migrating motor complex (MMC) - a cyclical pattern of motility in the stomach and small intestine that empties these gut regions in preparation for the next meal and removes luminal debris (6). MMC cycles vary in duration from 2-4 hours and are interrupted by feeding (13). Peak levels of motilin in humans were found to be elevated just before the start of gastric origin phase III contractions, leading to the suggestion that motilin is itself a regulator of gastric contractions (4). Supporting this idea, exogenous motilin administration induced MMC gastric phase III contractions in the Asian house shrew (14) and man (2).

In the post prandial state, the MMC and the cyclical variation of plasma motilin levels are disrupted. Neither the underlying cause of the cyclical slow motilin waves in the fasting state nor the stimuli regulating postprandial motilin levels are well understood. Following food ingestion, studies have reported that motilin levels are variously: increased by fat, decreased by glucose, either increased or decreased by protein or mixed meals, and increased by bile acids and duodenal acidification (4). In human duodenal organoids, stimuli of motilin release *in vitro* include fatty acids, bile acids and low pH (15).

Our relatively poor understanding of the factors regulating motilin levels is attributable to the lack of reliable commercially available motilin assays and rodent models. Most publications assessing motilin levels have employed a radioimmunoassay (RIA) (13), which appears reliable but is not commercially available, and like other antibody-based methods is potentially prone to antibody cross-reactivity. Liquid chromatography with tandem mass spectroscopy (LC-MS/MS) has proved capable of detecting motilin in human gastric and duodenal biopsies (8), and in supernatants from human duodenal organoids (15), raising the possibility of developing a quantitative LC-MS/MS method to assay motilin in human plasma. One published LC-MS/MS method for measuring motilin concentrations used rat plasma spiked with porcine motilin as the reference standard (16) but had selectivity and sensitivity problems with a detection limit of 10 ng mL^-1^, considerably above the reported motilin levels measured in humans which are in the region of 0.2-0.5 ng mL^-1^ (13, 17). A more recent study used LC-fluorescence to detect motilin in human plasma, but the reliability of this methodology is questionable (18) as borne out by their conclusion that endogenous motilin concentrations are in the region of 6-8 ng mL^-1^, substantially higher than reported by RIA.

In this study, we developed a quantitative LC-MS/MS method for measuring motilin in human plasma, which we used to characterise motilin levels during 4 hours of fasting after an overnight fast and in response to a subsequent liquid meal in healthy human volunteers. As this bioanalytical technique is readily multiplexable, it was simultaneously developed for the co-measurement of insulin, C-peptide, GIP (1–42) and GIP(3-42). Bioactive GIP(1-42) only circulates for a short time before inactivation by dipeptidyl peptidase IV to produce inactive GIP(3-42) (19); the use of targeted LC-MS/MS provides the opportunity to quantify both GIP isoforms simultaneously due to their difference in molecular weight, as described in previous LC-MS/MS protocols (20–22). Immunoassay analysis was additionally performed for active ghrelin and total GIP, to correlate ghrelin and motilin profiles, and compare the different methods for GIP detection.

## Methods

### Materials

Unless stated otherwise all reagents were commercially sourced and used as supplied. HPLC grade methanol, acetonitrile and water (Fisher Scientific, Loughborough, UK) were used for all analyses. Reagent grade bovine serum albumin (BSA), formic acid, and acetic acid (Sigma Aldrich, Poole, UK) were used for extraction methods.

Reference standards for motilin, GIP(1-42), GIP(3-42), C-peptide (Bachem), and Actrapid (human insulin; NovoNordisk) were stored at -20°C as 1 mg mL^-1^ solutions, in 20% methanol/0.1% formic acid/0.1% BSA (aq).

Heavy labelled sequences of motilin (FVPI-[U-13C9,15N-Phe]-TYGE[U-13C6,15N-Leu]-QRMQEKERNKGQ-acid) and GIP(1-42) (YAEGT-[U-13C9,15N-Phe]-ISDYSIAMDKIHQQDFVNW-[U-13C6,15N-Leu]- [U-13C6,15N-Leu]-AQKGKKNDWKHNITQ-acid) were synthesised to order (Cambridge Research Biochemicals Ltd, Billingham, UK) for use as internal standards, and bovine insulin (Sigma Aldrich, Poole, UK) was used as an analogue internal standard for insulin and C-peptide. These were stored as a 1 mg mL^-1^ reference solution, diluted in 20% methanol/0.1% formic acid/0.1% bovine serum albumin (aq).

Pooled human plasma potassium EDTA anticoagulant (K_2_EDTA) (BioIVT, West Sussex, UK) from healthy controls was used as a matrix for the preparation of calibration standards during the clinical sample analysis.

All experimental procedures were performed on an M-Class Acquity (Waters, Milford, US) microflow LC system coupled to a TQ-XS triple quadrupole mass spectrometer with an ionKey source (Waters), using selected reaction monitoring (SRM) transitions for each peptide. Data were processed on TargetLynx XS (v 4.2, Waters) and statistical analysis was performed using Graphpad Prism (version 9).

### Study design

Ten healthy volunteers aged 18–65 years old were recruited onto the GutHHD study approved by the NHS research ethics committee (22/ES/0021), and all participants gave full written consent. All subjects were free from chronic diseases with no prior diagnosis of anaemia, endocrine or gastroenterological condition. Participants were either taking no medication or were stable on medication that was considered unlikely to interfere with the results of the study. One week bowel habit diaries were taken post study day to confirm regular bowel habit.

Participants attended the Clinical Research Facility at Addenbrooke’s Hospital on a single occasion following an overnight fast. The evening before each visit, participants were instructed to prepare a standardized meal aiming to consist of 15% protein, 30% fat and 55% carbohydrate. After the evening meal, participants were allowed free access to water but were asked to avoid food, caffeinated and calorie-containing drinks overnight from midnight prior to the study visit. Water was permitted until 1 hour before arriving at the research facility.

On initiation of the study visit, participants continued to fast for 4 hours with blood samples being collected at 15 minute intervals. Immediately after collection of the 240 min sample, a standard mixed meal was ingested and blood samples taken at baseline just before the meal and at further time points (15, 30, 45, 60, 90, 120 min) after the meal. The mixed meal consisted of 237 mL Ensure plus, a balanced nutritional supplement containing 355 kcals (1497 kJ) including 12 g fat, 15 g protein and 48 g carbohydrate. It also contains vitamins and minerals, not limited to and including A, B1, B2, B6, B12, calcium, iron, iodine, magnesium and zinc. The meal was consumed within a 5 min time frame and participants followed this immediately with 250 mL of water. At each time point participants were asked to mark their hunger score between 0 and 10 cm on a visual analogue scale.

At each time point blood samples were collected into EDTA, placed immediately on ice and centrifuged for 10 minutes at, 3500 x *g* at 4°C. The plasma layer was aliquoted to a clean sample tube, snap frozen on dry ice and stored at -70°C until required for analysis. Additional samples for ghrelin analysis were taken at each time point, by adding blood immediately to tubes containing the protease inhibitor AEBSF, placed on wet ice and centrifuged as per EDTA samples; 250 μL plasma aliquots were added to 50 μL 1M hydrocholic acid before being snap frozen and stored at -70°C.

The initial fasting time point also had samples screened for full blood count, urea and electrolytes, TSH, HbA1c and liver function tests, performed at Addenbrookes hospital clinical laboratories.

### Immunoassays

Validation of the GIP measurements was performed using 32 postprandial samples from the first 7 participants of the GutHDD study, and 61 stored samples from 4 participants of a previous study (23) in the fasting state and following consumption of either a mixed liquid meal or oral glucose tolerance test. Samples were analysed using a total GIP ELISA (Mercodia), which is reported to detect both GIP(1-42) and GIP(3-42). Of the stored samples from the previous study, 30 were below the detection limit for either GIP(1-42) or GIP(3-42) in the LC-MS/MS analysis and were excluded from the LC-MS/MS vs ELISA comparison. Ghrelin was measured using a Millipore Active Ghrelin kit. Glucose was measured from EDTA samples in fed samples.

### LC-MS/MS

For extraction, 100 µL of the sample was precipitated with 500 µL 80% acetonitrile containing 0.2 ng/mL of each of bovine insulin, motilin internal standard (IS) and GIP IS, and vortex mixed. Samples were centrifuged at, 3000 x *g* for 5 minutes. The supernatant was transferred to an Eppendorf Lo-bind plate and evaporated under nitrogen on a Biotage SPE dry system at 40°C. The samples were reconstituted in 200 µL 20% acetonitrile/0.1% formic acid (aq) and gently vortex mixed. The samples were transferred onto an Oasis HLB µElution SPE plate and gentle positive pressure was applied on a SPE manifold. The samples were washed with 200 µL 0.1% formic acid (aq) and 200 µL 5% methanol/1% acetic acid (aq). Samples were eluted into a clean QuanRecovery plate, with two aliquots of 30 µL 60% methanol/10% acetic acid (aq). Samples were diluted with 75 µL 0.1% formic acid (aq) and centrifuged at, 3000 x *g* for 5 minutes, prior to injection onto the LC system.

Each extracted sample (10 μL) was injected onto a nanoEase M/Z Peptide BEH C18 Trap Column (130Å, 5 μm, 300 μm X 50 mm, Waters) at 15 μL min^-1^ for a 3 minute load, with mobile phases set to 90% A (0.1% formic acid in water) and 10% B (0.1% formic acid in acetonitrile). The iKey HSS T3 Separation Device (100Å, 1.8 μm, 150 μm X 50 mm, Waters) was set at 45°C and the analytes were separated over a 13-minute gradient from 10% to 55% B, at a flow rate of 3 μL min^-1^. The iKey was flushed for 3 minutes at 85% B before returning to initial conditions, resulting in an overall run time of 20 minutes. Electrospray ionisation was performed, with a capillary voltage of 3 kV, collision gas flow was at 0.14 mL/min and the ionKey source temperature was 150°C. Precursor and product ions used for the analysis are given in **Table 1**.

**Table 1:** Targeted transitions used for each analyte.

Analytes	Precursor ion	Product ion	Collision Energy (eV)
Motilin	540.6	748.4	27
GIP (1-42)	831.6	207.05	27
GIP (3-42)	792.4	841.03	20
Insulin	1162.5	226.2	35
C-peptide	1007.4	927.5	30
Bovine insulin (Internal standard)	956.3	1120.8	22
Motilin IS	544.6	613.5	18
GIP IS	1002	1194	25

### Data analysis

For all analytes, the chromatography peak areas were integrated using an optimised TargetLynx XS protocol. These values were exported to Graphpad Prism, where a linear 1/x^2^ least squares regression was applied to calibration samples, to generate concentrations of the quality control and study samples. Concentrations of insulin and C-peptide in some of the fed samples were above the top point of the calibration curve, and were calculated by extrapolation of the regression line. Graphical plots and statistical analysis presented were performed in Jupyter notebook using Python 3.12.0 with packages pandas, numpy, seaborn, Matplotlib, scikit-learn and scipy.

Values are reported as mean with standard deviation unless otherwise stated, with p=0.05 used as a level of significance on statistical tests. For visualization plots and modelling, if a hormone was below the limit of detection, its value was set at the limit of detection, which for motilin was 20 pg mL^-1^.

Correlation analysis was performed using linear regression with r-squared coefficient of determination reported. Paired t tests were used for comparing fed with fasting motilin values.

For comparison of motilin with ghrelin and hunger scores, hormone levels and hunger scores were standardized using a minimum-maximum scaling approach, where the highest value of each hormone/hunger score was converted to 1 for each participant, and 0 for the lowest value. This was performed using scikit-learn MinMaxScalerfunction.

Motilin fluctuations in the fasting state were fitted with sine wave curves. Starting parameters for displacement and amplitude were calculated from maximum and minimum fasting motilin levels for each participant. The first two peaks in each participant’s fasting values were visualized by eye and used to calculate starting parameters for frequency and phase. Final parameters were then calculated and plotted using the scipy package function curve optimisation fit using least square method.

## Results

### Motilin assay development and characterisation

**Figure 1:**
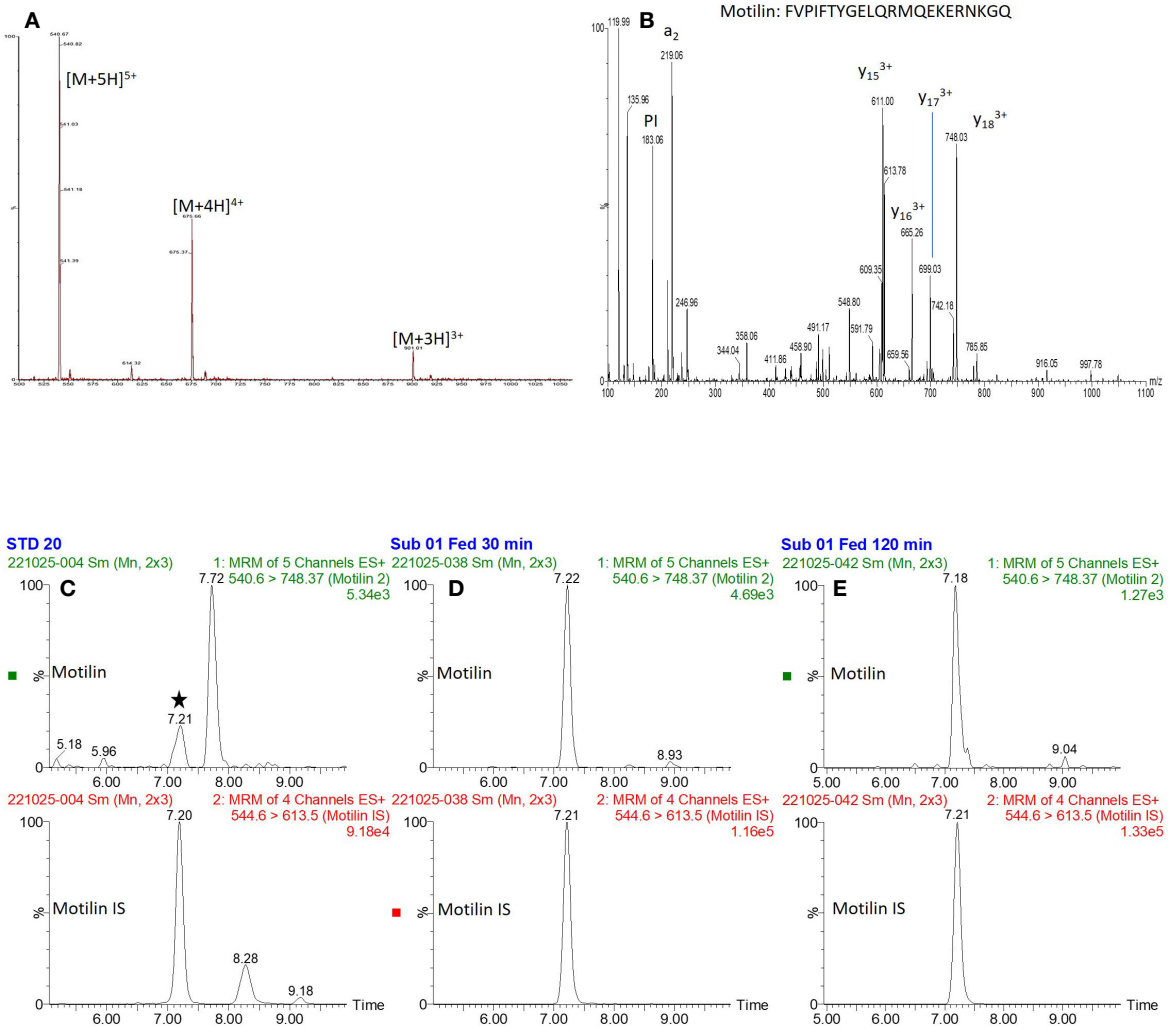
Precursor and product ion spectra for motilin, and example chromatograms. **(A, B)**: Precursor ion spectrum **(A)** for motilin showing multiple charge states, and product ion spectrum **(B)** of the [M+5H]^5+^ precursor ion. **(C–E)**: Extracted ion chromatograms of motilin and its internal standard (retention time of ~7.2 minutes) for **(C)**: 20 pg mL^-1^ standard, **(D)**: Subject 1, 30 minute fed time point and **(E)**: subject 1, 120 minute fed time point.

Precursor and product ion spectra for motilin are shown in **Figure 1**, together with example chromatograms for motilin and its internal standard. Chromatograms for all analytes (motilin, GIP(1-42), GIP(3-42), insulin and C-peptide) in a QC sample are shown in **Supplementary Figure 1**. A precision and accuracy analysis was performed over the concentration range of 20-2000 pg mL^-1^ for each analyte. Calibration standards and quality control samples were prepared in pooled human plasma (n = 6 for QCs), extracted for analysis, and run on the ionKey LC-MS/MS method. Peak area ratios were calculated against the corresponding internal standards for motilin and GIP, or against bovine insulin for insulin and C-peptide. The results for all analytes showed suitable calibration curves (**Supplementary Figure 2**) and the standard addition approach was applied to calculate concentrations of motilin, GIP(1-42), GIP(3-42), insulin and C-peptide, compensating for endogenous levels in the pooled human plasma. The QC sample concentrations were within 25% %RE and %CV (**Table 2**) for all analytes, confirming that the assay was suitable for parallel measurement of motilin, GIP, insulin and C-peptide in human plasma samples.

**Table 2:** Precision (%CV) and accuracy (%RE) of all analytes in n = 6 quality control samples, prepared at four concentration levels.

Spiked Concentration (pg/mL)	30	60	150	1500
Motilin				
%CV	20.4	21.1	10.4	13.8
%RE	-7.0	1.2	10.1	23.7
GIP (1-42)				
%CV	14.0	28.1	19.1	9.8
%RE	9.8	17.5	21.4	-10.4
GIP (3-42)				
%CV	12.0	15.7	18.8	14.1
%RE	13.0	17.9	19.0	18.1
Insulin				
%CV	19.5	13.5	13.0	15.8
%RE	-1.7	-14.9	-10.6	-19.4
C-peptide				
%CV	18.0	21.3	15.7	6.6
%RE	-7.5	-12.6	-12.2	2.8

Due to the presence of endogenous peptides in the pooled human plasma a standard addition approach was used to confirm the total peptide concentration for all analytes except GIP (1-42).

### Motilin, ghrelin and hunger during a 4 hour fast

Blood samples were collected from 10 healthy volunteers during a 4 hour fast and following a liquid test meal. Volunteer demographics are given in **Table 3**. Bowel habit diaries were returned by 9 participants and showed normal patterns with an average stool form of 4.2 on the Bristol Stool Chart Form Scale and a mean of 1.3 motions per day.

**Table 3:** Table of demographics for 10 participants in the study, displayed as mean and standard deviation unless otherwise stated.

Gender (male, %)	7.0	(70.0)
Age	38.6	(11.1)
BMI	24.5	(2.7)
Weight (kg)	76.2	(11.6)
height (cm)	176.3	(7.6)
Waist Cir (cm)	84.8	(10.2)
Hb (g/L)	138.4	(11.7)
TSH (U/L)	1.6	(0.4)
HbA1c (mmol/mol)	33.0	(2.9)
ALT (IU/L)	21.6	(5.5)

**Figure 2:**
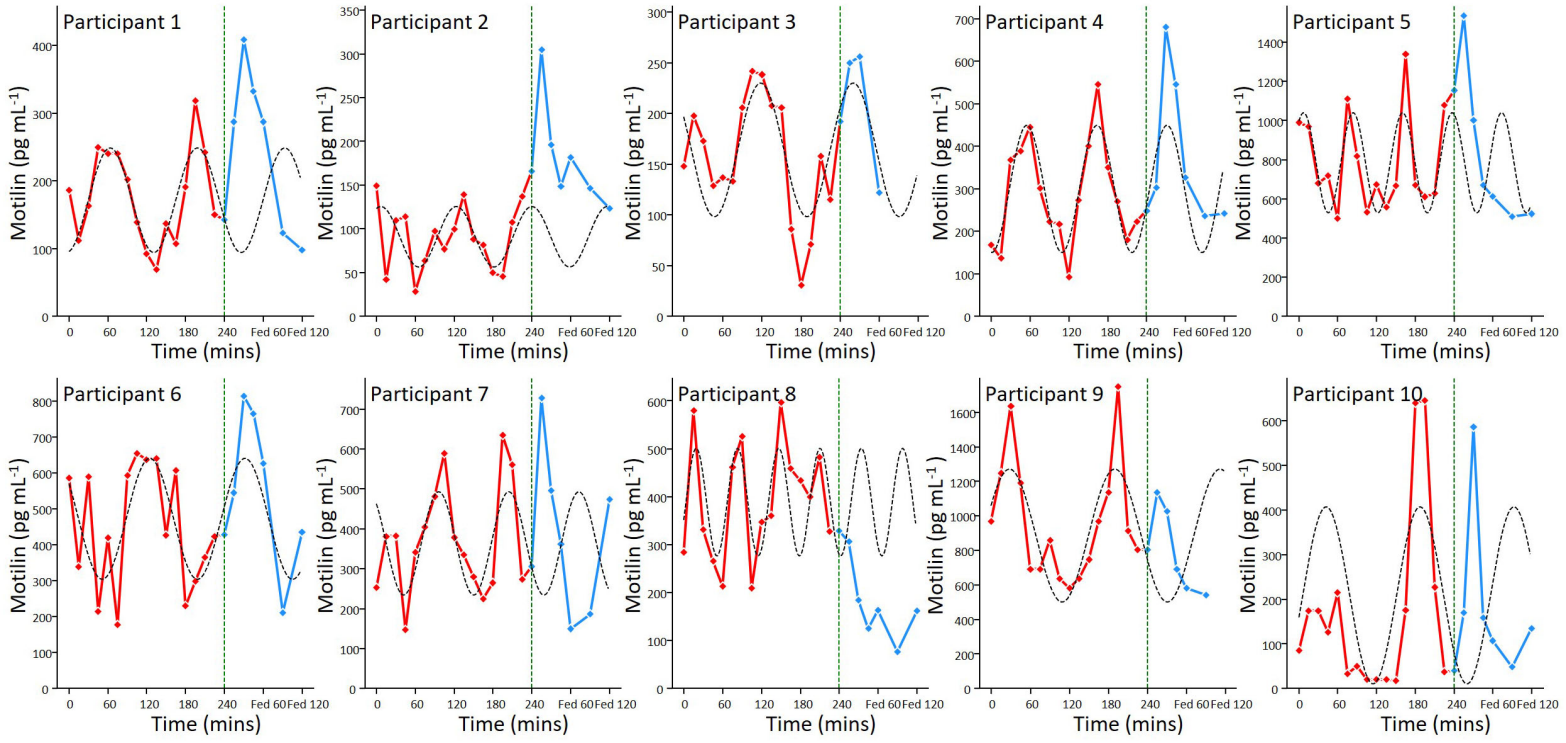
Motilin fluctuations in the fasting and fed state. Individual motilin profiles for all 10 participants, with samples taken every 15 minutes during a 4 hour prolonged fast (red) and then 15, 30, 45, 60, 90, 120 minutes following a mixed meal (blue). The meal was ingested immediately after the 240 min time point, as indicated by the dashed line. Sine waves fitted to the fasting data (dashed lines) have been extended beyond 240 min for illustration.

All participants had detectable levels of motilin in their fasting plasma samples, which exhibited large amplitude cyclical fluctuations (**Figure 2**), with peak concentrations averaging 7.8 (SD: 10.3) times the trough concentration in the same participant. Fluctuations in the fasting state for each participant were fitted with individual sine waves. The mean observed period for oscillations across participants was 110 mins (SD: 30), with trough of 185 (SD: 199) pg mL^-1^ and an amplitude (baseline to peak) of 504 pg mL^-1^ (SD: 311).

**Figure 3:**
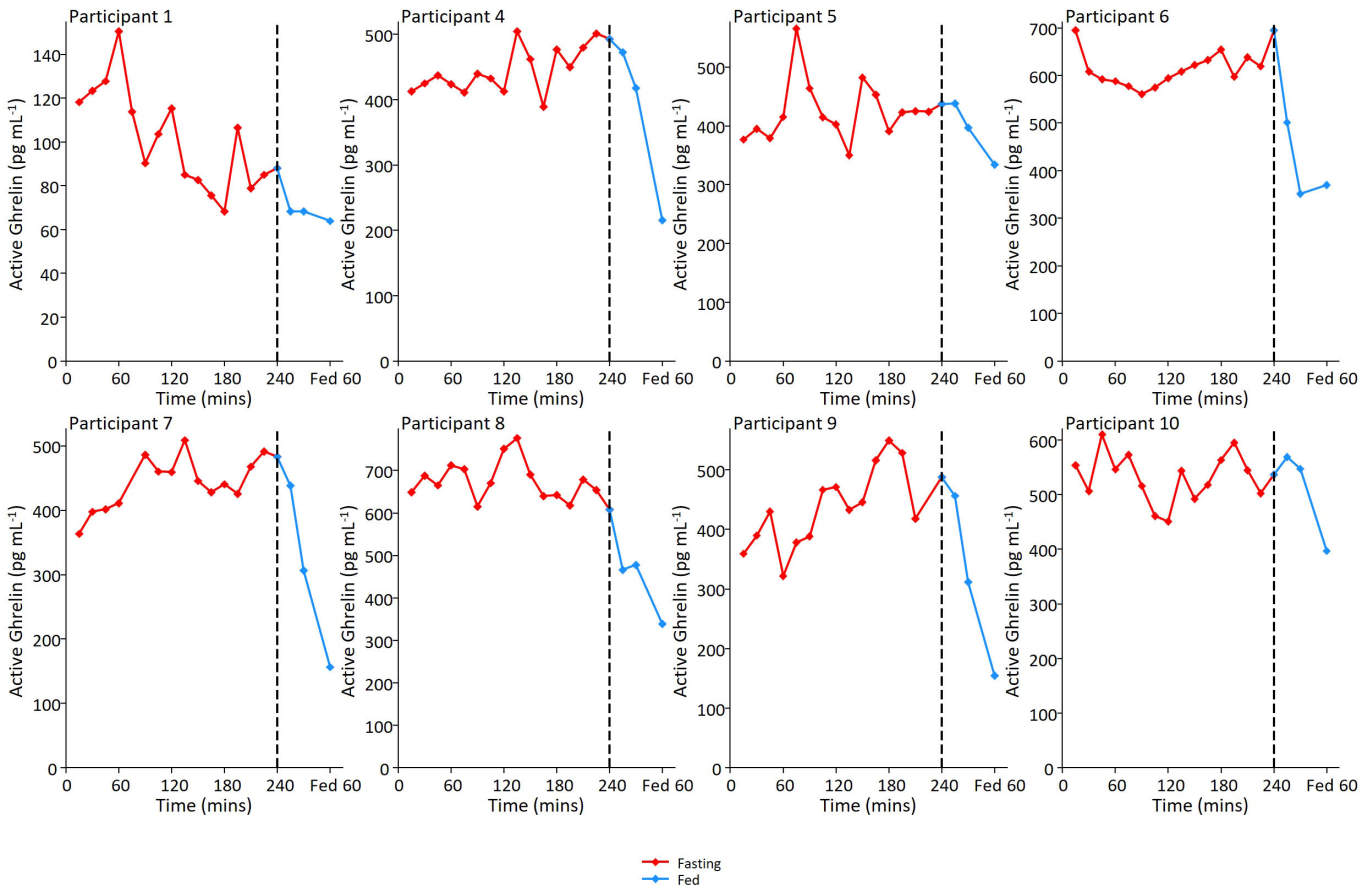
Ghrelin fluctuations in the fed and fasted state. Individual ghrelin profiles for 8 participants. In relation to motilin samples, ghrelin sampling was not performed at first time point, but was then performed on samples every 15 minutes during the remainder of the 4 hour prolonged fast and then 15, 30 and 60 minutes post meal ingestion.

Active ghrelin was measured in 8 participants from 15 mins into the protocol until the meal, and then 15, 30 and 60 mins post meal (**Figure 3**). Ghrelin was detectable at all time points and showed low amplitude fluctuations (peak:trough ratio = 1.5 (SD: 0.3)) in the fasting state with no clear periodicity.

**Figure 4:**
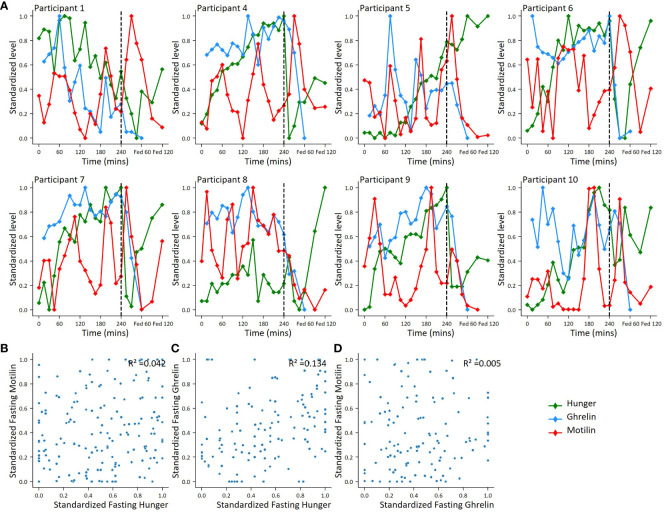
Correlations between motilin, ghrelin and hunger score fluctuations. **(A)**. For 8 participants with ghrelin and motilin profiles, individual plots showing motilin, ghrelin and hunger fluctuations across fasting and fed periods. Hormone levels and hunger scores standardized using minimum-maximum scaling converting highest value to 1 and lowest value to 0 across all time points. **(B–D)**. Scatter plots comparing standardised hormone level and hunger score. **(B)**: Standardized fasting motilin vs hunger score (n=10), **(C)**: Standardized fasting ghrelin vs hunger (n=8); **(D)**: Standardized fasting motilin vs standardized fasting ghrelin (n=8).

Motilin and ghrelin levels and hunger scores were scaled between zero and 1 across all time points to look for evidence of synchronisation (**Figure 4A**). Ghrelin and motilin did not appear to be synchronised with each other. Hunger scores appeared to peak before the mixed meal but were not clearly associated with increases in motilin or ghrelin levels. Analysis of the scaled data across all fasting time points revealed: no evidence of a relationship between motilin and hunger scores (r^2^ = 0.042); no evidence of relationship between ghrelin and motilin levels (r^2^ = 0.005), and limited evidence of a relationship between fasting ghrelin and fasting hunger scores (r^2^ = 0.134) (**Figures 4B–D**).

### Hormone levels following mixed liquid meal ingestion

For one participant, the post meal sampling was stopped early at 60 mins as they felt lightheaded and did not wish to continue; post meal samples for this participant have not been included in the mean data. Motilin levels following the mixed meal peaked at 15-30 mins in 9 out of 10 participants, interrupting the predicted continuation of oscillations from the fasting period (**Figure 2**). Across all participants, peak fed motilin values were significantly higher than concentrations immediately before the meal (p=0.0006). Peak maximum motilin levels after the meal were higher than the highest level reached in the fasting state in 7 participants, but across all 10 participants this was not significant (p=0.87).

**Figure 5:**
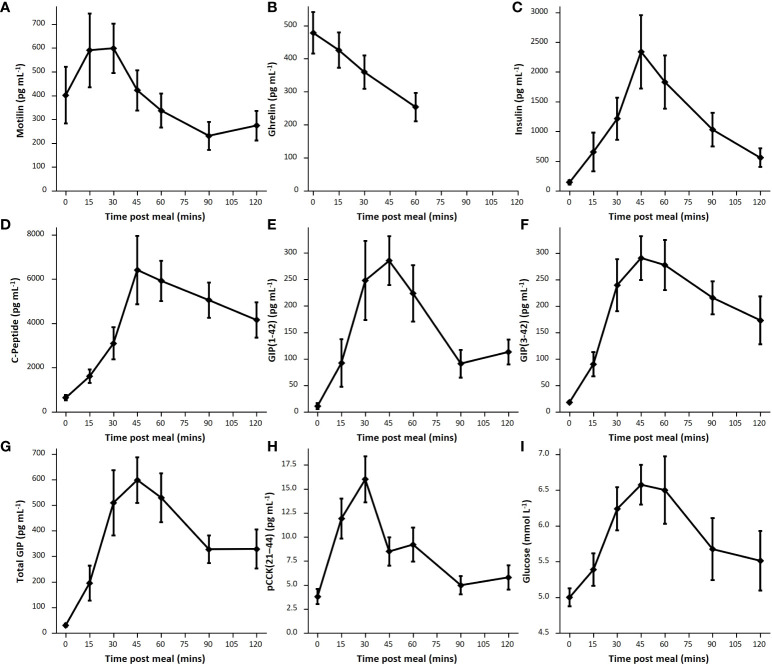
Postprandial hormone levels. Average hormone levels following mixed meal for each hormone, shown as mean and standard error. **(A)**: Motilin (n=9), **(B)**: Ghrelin (n=8), **(C)**: Insulin (n=9), **(D)**: C-peptide (n=9), **(E)**: GIP(1-42) (n=7), **(F)**: GIP(3-42) (n=9); **(G)**: Total GIP calculated as total sum of GIP(1-42) and GIP(3-42) (n=7); **(H)**: pCCK(21-44) (n=8). **(I)**: glucose (n=8).

The mixed meal stimulated a robust increase in GIP(1-42), GIP(3-42), C-peptide, insulin and glucose in all measured participants, within 45 minutes of meal ingestion (**Figure 5**). The LC-MS/MS method allowed separate analysis of GIP(1-42) and GIP(3-42), with GIP (3-42) observed at consistently higher levels than GIP(1-42), most likely due to its longer half-life in plasma. Ghrelin levels were suppressed after ingestion of the mixed meal (**Figure 3**, **Figure 5**). ProCCK (21–44) levels, reported previously in the same group of participants as a proxy for CCK concentrations (24), are shown in **Figure 5** for comparison of the time course of different hormonal changes after the liquid meal.

To validate the GIP method we compared LC-MS/MS measurements of GIP(1-42) and GIP(3-42) with total GIP concentrations measured by ELISA in a subgroup of postprandial samples from the current study (GutHDD) and stored samples from a previous study (23). Good agreement was observed between the two types of measurement (**Supplementary Figure 3**), confirming that the LC-MS/MS method is suitable for measuring GIP peptides in human plasma. LC-MS/MS has the additional advantage of distinguishing active GIP(1-42) from the inactive DPP4 degradation product GIP(3-42). However, the GIP ELISA has a better lower sensitivity limit (8 pg mL^-1^ for total GIP) compared with the current LC-MS/MS method (20 pg mL^-1^ for each individual peptide).

## Discussion

Fasting and postprandial motilin concentrations in human plasma are typically measured by RIA, but the application of the new LC-MS/MS method to quantify motilin levels will support future studies aimed at understanding the physiology of this gut hormone in healthy human participants, and how it is disturbed in metabolic and gastrointestinal conditions. An additional benefit to LC-MS/MS based analysis of motilin over an RIA approach is that the instrument measures the intact peptide directly – monitoring the [M+5H]^5+^ charge state of the peptide and a [M+3H]^3+^ charged y17 fragment. The selectivity achieved by targeting this “transition” compared with indirect immunoreactive measurements using antibodies ensures no cross-reactivities occur in LC-MS/MS based analyses. Despite these differences in bioanalytical approaches, the concentrations generated by RIA and LC-MS/MS are similar for fasted individuals – in the 100-1000 pg/mL concentration range. The LC-MS/MS method was capable of measuring much lower motilin concentrations than achievable by LC-fluorescence after derivatisation with a fluorescent tag (18), which only measured levels of around 7 ng/mL in a single timepoint of unknown fed/fasted state. In addition to the selectivity of the LC-MS/MS approach, the ability to detect multiple peptides in a single sample makes it an advantageous analytical method from the perspective of sample volume, as a single plasma sample of 100 μL was used to measure all the peptides in parallel. The measurement of GIP(1-42), GIP(3-42), insulin and C-peptide alongside motilin using the LC-MS/MS method, as well as active ghrelin by immunoassay, revealed the contrast between the plasma profiles of these different hormones, with motilin concentrations exhibiting cyclical variation in the fasting state and a small postprandial rise, compared with GIP, insulin and C-peptide which were low in the fasting state and substantially elevated after ingestion of the mixed liquid meal, and ghrelin which did not exhibit substantial fluctuations in the fasting state and was suppressed after feeding.

Fasting motilin concentrations oscillated in all subjects, with a mean peak-peak interval of 110 minutes. The measured motilin concentrations and oscillation patterns are similar to those reported in previous studies using RIA (13, 17). As reported by RIA, we observed motilin levels to rise in most subjects after the meal, with the post-prandial peak at 15-30 mins significantly higher than the value before the meal was given. There are discrepancies in the literature about which nutrients have the greatest impact on motilin secretion, with variable effects reported for carbohydrates, fats and protein (25, 26). Our previous studies using human duodenal organoids revealed that motilin secretion is directly stimulated by acidification, fatty acids and bile acids (15).

The individual motilin profiles show considerable variation across participants. Previous studies have reported that motilin can vary in certain gastrointestinal conditions, which were excluded in this study, and with increasing age (4). Within the limits of our observational number of 10, no significant evidence of an association between maximum motilin levels and age was found. As healthy participants would not normally fast for 4 hours in the morning after an overnight fast, the observed motilin fluctuations do not represent normal fasting/feeding patterns, and it is unknown how this extended fast would have influenced motilin fluctuations and MMCs. Our participants did not have manometry contractility measurements, so it is not possible to determine how the measured fluctuations related to gastric or duodenal origin contractions (13).

To enable interindividual comparisons and account for subjectivity in hunger scores, an individual-based minimum-maximum scaler was applied to motilin and ghrelin levels and hunger scores, to preserve fluctuations within the data but standardize scores and hormone levels to the same scale. This analysis did not reproduce previous reports that hunger scores vary in the fasting state alongside motilin level fluctuations (2). Key differences in our study, however, were the prolonged duration of the fast, using a minimum-maximum scalar in analysis, and that participants were expecting the arrival of the meal at the end of the fasting period. A weak association was observed between ghrelin and hunger scores, but our assessment of hunger was potentially confounded by the limitation that participants knew when the liquid meal would arrive, and the small number of participants within this observational study.

Following ingestion of the mixed liquid meal, we observed an increase in plasma concentrations of motilin, GIP, insulin, C-peptide and pCCK(21-44), and a fall in ghrelin. Whereas peak motilin and pCCK(21-44) levels were reached at 15-30 minutes, concentrations of GIP, insulin, glucose and C-peptide peaked slightly later at 45 minutes. One potential explanation for the earlier CCK and motilin peaks compared with GIP, despite all three hormones being released from the upper small intestine, relates to the composition of the mixed liquid meal. This contained 12g of fat which was pre-emulsified, making it potentially readily digestible by gastric lipases and thus able to stimulate early release of motilin and CCK. The majority of the meal was carbohydrate (47g, including 16g of free sugars), which may be absorbed over a longer time frame providing ongoing stimulation of GIP and insulin release, as reflected by the matching time course of plasma glucose.

The GIP component of the multiplexed assay was able to quantify postprandial GIP(1-42) and GIP(3-42) concentrations, although could not quantify low levels of both peptides in the fasting state in all participants. Results using the LC-MS/MS method correlated well with total GIP concentrations measured by an ELISA that detects both GIP(1-42) and GIP(3-42) with 100% cross-reactivity. Insulin and C-peptide measurements performed in the multiplexed LC-MS/MS assay were similar to those detected in healthy subjects in previous studies (23, 27).

To conclude, a multiplexed LC-MS/MS method for the measurement of motilin, GIP(1-42), GIP(3-42), insulin and C-peptide in human plasma was successfully developed. When applied to clinical samples from healthy volunteers, the assay confirmed previous reports of motilin fluctuations in the fasting state, followed by a small post-prandial rise. As LC-MS/MS does not require high affinity antibodies and quantifies the true motilin sequence, it is not prone to assay cross-reactivities that are typical of immunoassay approaches. This LC-MS/MS method can be used as a baseline for further clinical studies into motilin physiology in human volunteers in human health and different gastrointestinal disease states, and can be readily multiplexed within the same assay for measurement of other hormones.

## Data availability statement

The raw data supporting the conclusions of this article will be made available by the authors, without undue reservation.

## Ethics statement

The studies involving humans were approved by East of Scotland Research Ethics Service (EoSRES) REC reference: 22/ES/0021. The studies were conducted in accordance with the local legislation and institutional requirements. The participants provided their written informed consent to participate in this study.

## Author contributions

RF: Conceptualization, Data curation, Formal analysis, Investigation, Methodology, Writing – original draft. CB: Conceptualization, Data curation, Formal analysis, Investigation, Methodology, Project administration, Visualization, Writing – original draft. RK: Conceptualization, Data curation, Methodology, Supervision, Writing – review & editing. FR: Conceptualization, Funding acquisition, Methodology, Supervision, Writing – review & editing. FG: Conceptualization, Funding acquisition, Methodology, Supervision, Writing – review & editing.
