# Supplementary material for: Motilin fluctuations in healthy volunteers determined by liquid chromatography mass spectrometry
Source: Front Endocrinol (Lausanne). 2024 Mar 13;15:1348146. doi: 10.3389/fendo.2024.1348146 (PMC10965782; doi:10.3389/fendo.2024.1348146)
Supplement: Supplementary file 1 [file Image_1.pdf]

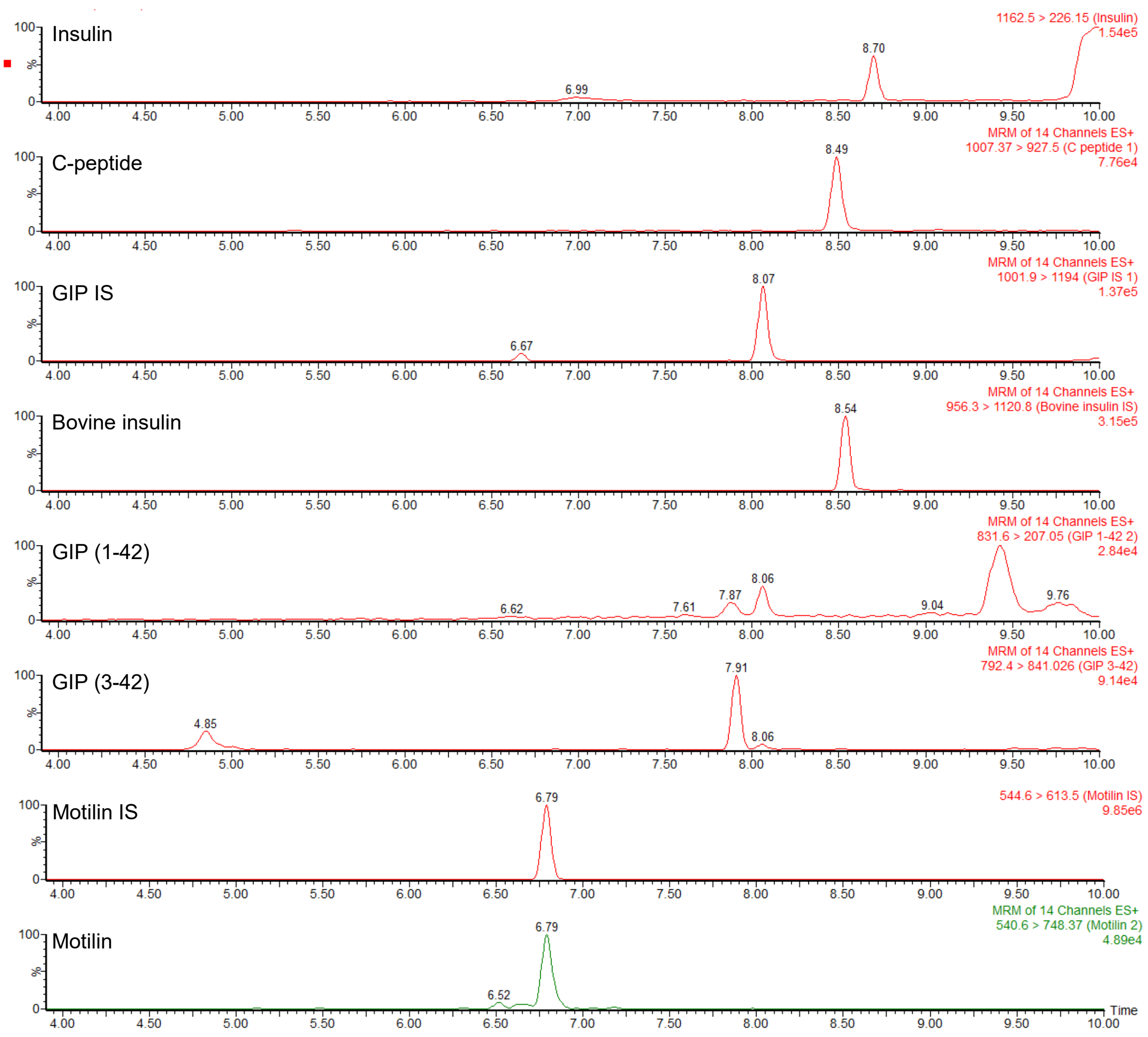

Supplementary Figure 1: Chromatograms for all analytes (and internal standards) measured in an extracted 500 pg/mL QC sample.

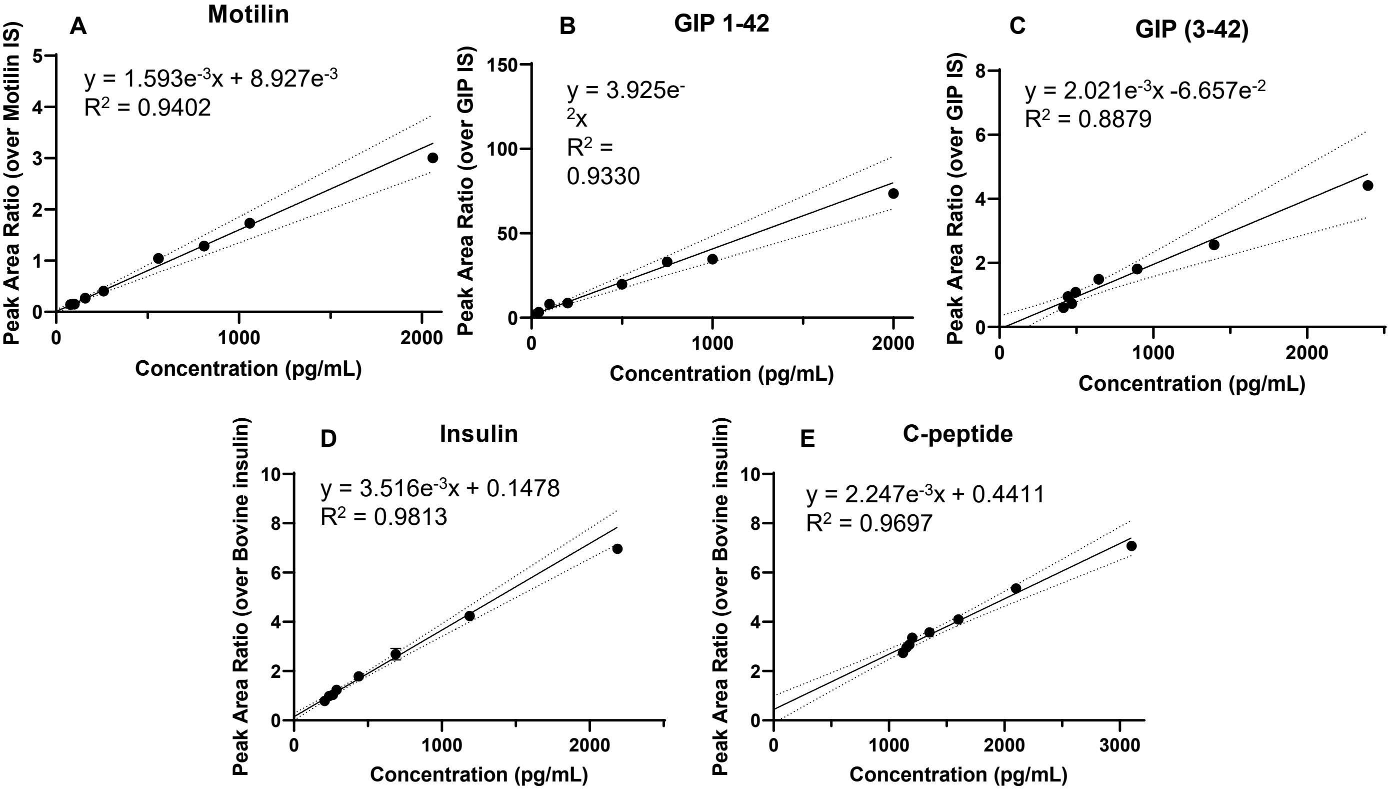

Supplementary figure 2:
Calibration curves for each analyte detected with LC-MS/MS.

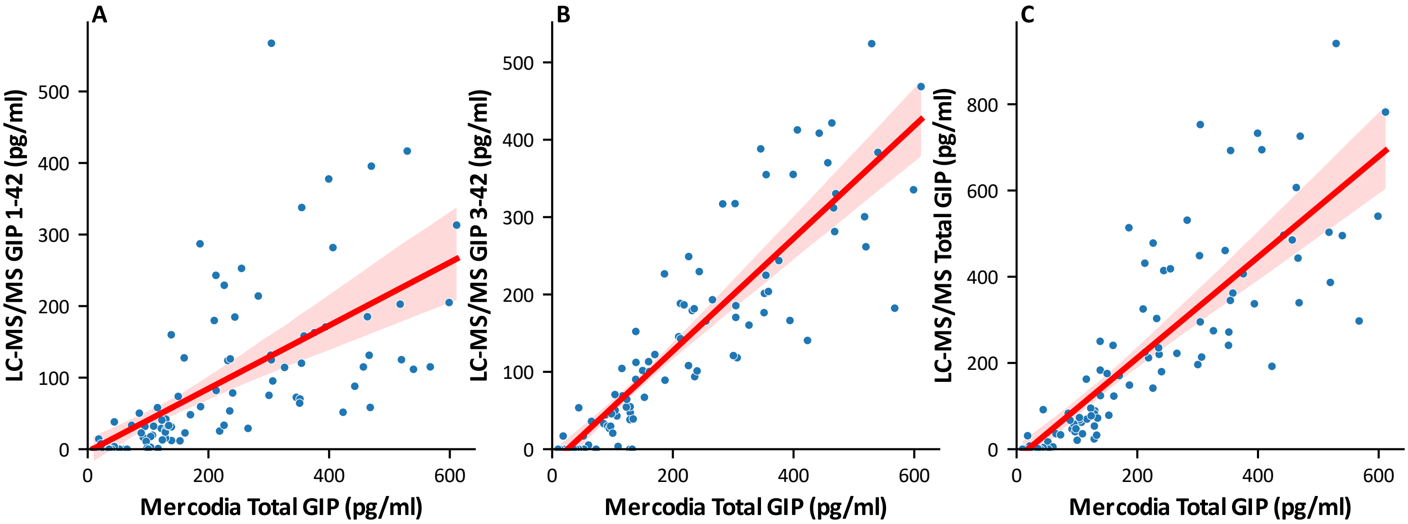

Supplementary Figure 3: Correlation curves for LC-MS/MS detected GIP vs ELISA Mercodia total GIP (n=93). A: GIP(1-42) vs ELISA total GIP; B: GIP(3-42) vs ELISA total GIP; C: Sum of LC-MS/MS GIP(1-42) and GIP (3-42) vs ELISA total GIP
